# Gradual enhancement of corticomotor excitability during cortico-cortical paired associative stimulation

**DOI:** 10.1038/s41598-022-18774-9

**Published:** 2022-08-29

**Authors:** Sonia Turrini, Francesca Fiori, Emilio Chiappini, Emiliano Santarnecchi, Vincenzo Romei, Alessio Avenanti

**Affiliations:** 1 Centro Studi e Ricerche in Neuroscienze Cognitive, Dipartimento di Psicologia, Alma Mater Studiorum – Università di Bologna, Campus di Cesena. Via Rasi Spinelli 176, 47521 Cesena, Italy; 2 Precision Neuroscience and Neuromodulation Program, Gordon Center for Medical Imaging, Massachusetts General Hospital and Harvard Medical School, Boston, MA USA; 3 Present Address: NeXT: Unità di ricerca di Neurofisiologia e Neuroingegneria dell’Interazione Uomo-Tecnologia, Università Campus Bio-Medico, Rome, Italy; 4 Present Address: Institut für Klinische und Gesundheitspsychologie, Universität Wien, Wien, Austria; 5 IRCCS Fondazione Santa Lucia, Rome, Italy; 6 Centro de Investigación en Neuropsicología y Neurociencias Cognitivas, Universidad Católica del Maule, Talca, Chile

**Keywords:** Motor control, Neural circuits, Sensorimotor processing, Synaptic plasticity

## Abstract

Cortico-cortical paired associative stimulation (ccPAS) is an effective transcranial magnetic stimulation (TMS) method for inducing associative plasticity between interconnected brain areas in humans. Prior ccPAS studies have focused on protocol’s aftereffects. Here, we investigated physiological changes induced “online” during ccPAS administration. We tested 109 participants receiving ccPAS over left ventral premotor cortex (PMv) and primary motor cortex (M1) using a standard procedure (90 paired-pulses with 8-ms interstimulus interval, repeated at 0.1 Hz frequency). On each paired-pulse, we recorded a motor-evoked potential (MEP) to continuously trace the emergence of corticomotor changes. Participant receiving forward-ccPAS (on each pair, a first TMS pulse was administered over PMv, second over M1, i.e., PMv-to-M1) showed a gradual and linear increase in MEP size that did not reach a plateau at the end of the protocol and was greater in participants with low motor threshold. Participants receiving reverse-ccPAS (i.e., M1-to-PMv) showed a trend toward inhibition. Our study highlights the facilitatory and inhibitory modulations that occur during ccPAS administration and suggest that online MEP monitoring could provide insights into the malleability of the motor system and protocol’s effectiveness. Our findings open interesting prospects about ccPAS potential optimization in experimental and clinical settings.

## Introduction

Cortico-cortical paired associative stimulation (ccPAS) is an effective transcranial magnetic stimulation (TMS) method for inducing associative plasticity between interconnected brain areas in humans^1–4^, based on the Hebbian principle of spike-timing-dependent plasticity (STDP)^5–7^. The ccPAS protocol consists in the repeated application of pairs of TMS pulses over two interconnected brain sites^1–4,7–15^, using an optimal interstimulus interval (ISI) between the pulses so that, for each pair, the first pulse administered over the first site (containing “pre-synaptic neurons”, according to the Hebbian principle^5^) would induce an activation spread reaching the second site (containing “post-synaptic neurons”) immediately before/simultaneously with the administration of a pulse over that site. This pre- and post-synaptic coupling mimics patterns of neural stimulation instrumental for achieving STDP^6,7^, thus enhancing (or weakening) the strength of the neural pathway connecting the stimulated brain areas^1–4,7–15^. Indeed, studies have reported that ccPAS induces changes in functional^13,14^ and effective^2,3,12,13^ connectivity of the targeted networks, as well as behavioral effects both in the motor^1,11,15^, visual^9,10,16,17^ and executive functions^8^ domains, suggesting that ccPAS could be a useful tool for investigating and changing behavior following plastic ‘re-wiring’ of the human connectome.

Notably, prior studies have mostly focused on physiological and behavioral aftereffects of ccPAS^1–3,8–14^, without clarifying whether and how plastic changes build up already during protocol administration. Addressing this issue is the main goal of the present study. Importantly, clarifying the dynamics of physiological changes “online” during ccPAS administration may provide insights into the optimal duration of the protocol. Moreover, while prior studies have suggested that interindividual differences in motor excitability predict sensitivity to exogenous manipulations of STDP^11,18^, whether individual’s resting motor threshold (rMT) predicts plastic changes during the administration of ccPAS is a relevant and yet largely unexplored issue.

To fill these gaps, we administered ccPAS over a premotor-motor circuit encompassing the left ventral premotor cortex (PMv) and the left primary motor cortex (M1), while continuously monitoring changes in corticomotor excitability via motor-evoked potentials (MEPs) recording. Indeed, because M1 was targeted using suprathreshold intensity, on each pulse a MEP was recorded in the contralateral (right) first dorsal interosseous of participants’ hand (see Methods for details).

The PMv-M1 is a hierarchically organized neural network primarily involved in fine motor control of sensory-guided actions such as grasping and manipulating objects^19,20^, but has also been implicated in several other functions including action imitation^21,22^, processing of observed actions^23–25^ and action-related language^26–28^. Of particular relevance to the present research, prior studies have established the temporal properties of the PMv-to-M1 pathway, by showing that a conditioning TMS pulse over PMv results in a modulation of MEPs induced by a second pulse over M1 when an ISI of 8 ms is used^29,30^. Accordingly, previous ccPAS studies targeting the PMv-to-M1 pathway have selected an 8-ms ISI to repeatedly and coherently couple pre- and post-synaptic activity—optimal for inducing STDP^2,11,14^.

**Figure 1:**
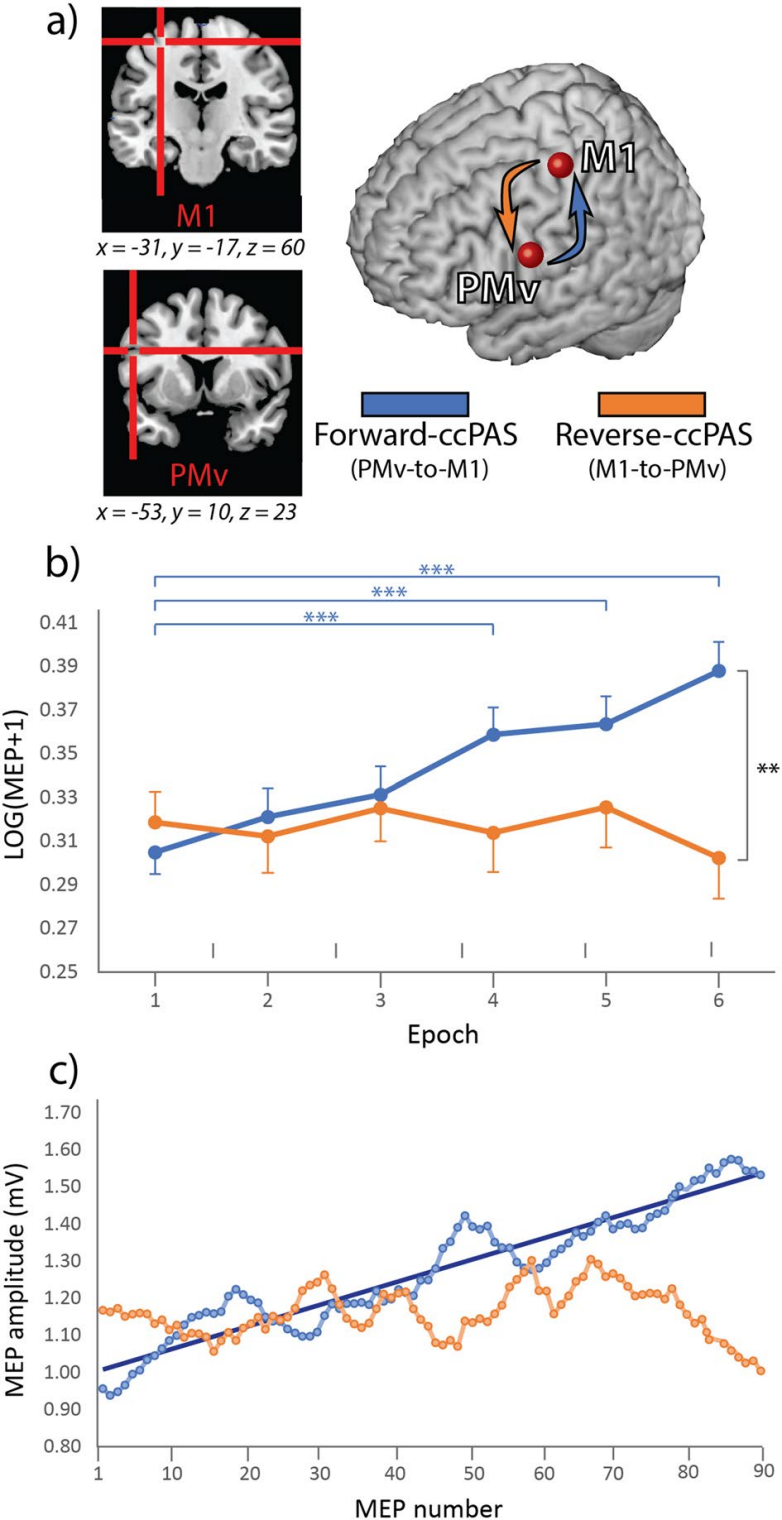
Targeted brain sites and MEP changes during ccPAS. (**a**) Mean Talairach coordinates of the targeted cortical sites reconstructed using MRIcron. (**b**) Changes in mean MEPs across Epochs. Error bars denote s.e.m. Asterisks indicate significant post-hoc comparisons: ∗∗*P* ≤ 0.01; ∗∗∗*P* ≤ 0.001. (**c**) Gradual changes in MEP size at the single-trial level.

Building on this prior work, we designed a ccPAS protocol consisting in 90 pairs of TMS pulses delivered at 0.1 Hz, adopting an 8-ms ISI to induce STDP in the PMv-to-M1 pathway. Participants were randomly divided into two groups (Fig. 1a): premotor-motor ‘forward-ccPAS’, i.e., a protocol aimed at enhancing the hierarchical organization of the circuit, where PMv conveys signals to M1 for motor command implementation, by repeatedly stimulating PMv before M1, or ‘reverse-ccPAS’, in which the order of the pulses on each pair was reversed-i.e., M1 stimulation was followed by PMv stimulation. Based on the Hebbian rule^5–7^, forward-ccPAS should induce long-term potentiation-like enhancement of the PMv-to-M1 pathway, resulting in increased corticomotor excitability, whereas reverse-ccPAS would weaken that pathway, resulting in decreased corticomotor excitability.

## Results

Figure 1b shows changes in corticomotor excitability during the ccPAS protocol in the two groups (see also Table S1). The Protocol (forward-ccPAS, reverse-ccPAS) x Epoch (1–6) ANOVA on MEP amplitudes revealed a significant main effect of Epoch (*F*_5,535_ = 8.25; *P* < 0.001; *η*_*p*_^*2*^ = 0.08), which was qualified by a significant Protocol x Epoch interaction (*F*_5,535_ = 13.06; *P* < 0.001; *η*_*p*_^*2*^ = 0.11). Tukey’s post-hoc tests showed that forward-ccPAS induced a clear increase in MEP amplitudes over time, significant from the fourth epoch onwards (all *P* < 0.001), while MEPs during reverse-ccPAS did not show consistent changes across epochs (all *P* ≥ 0.45).

**Figure 2:**
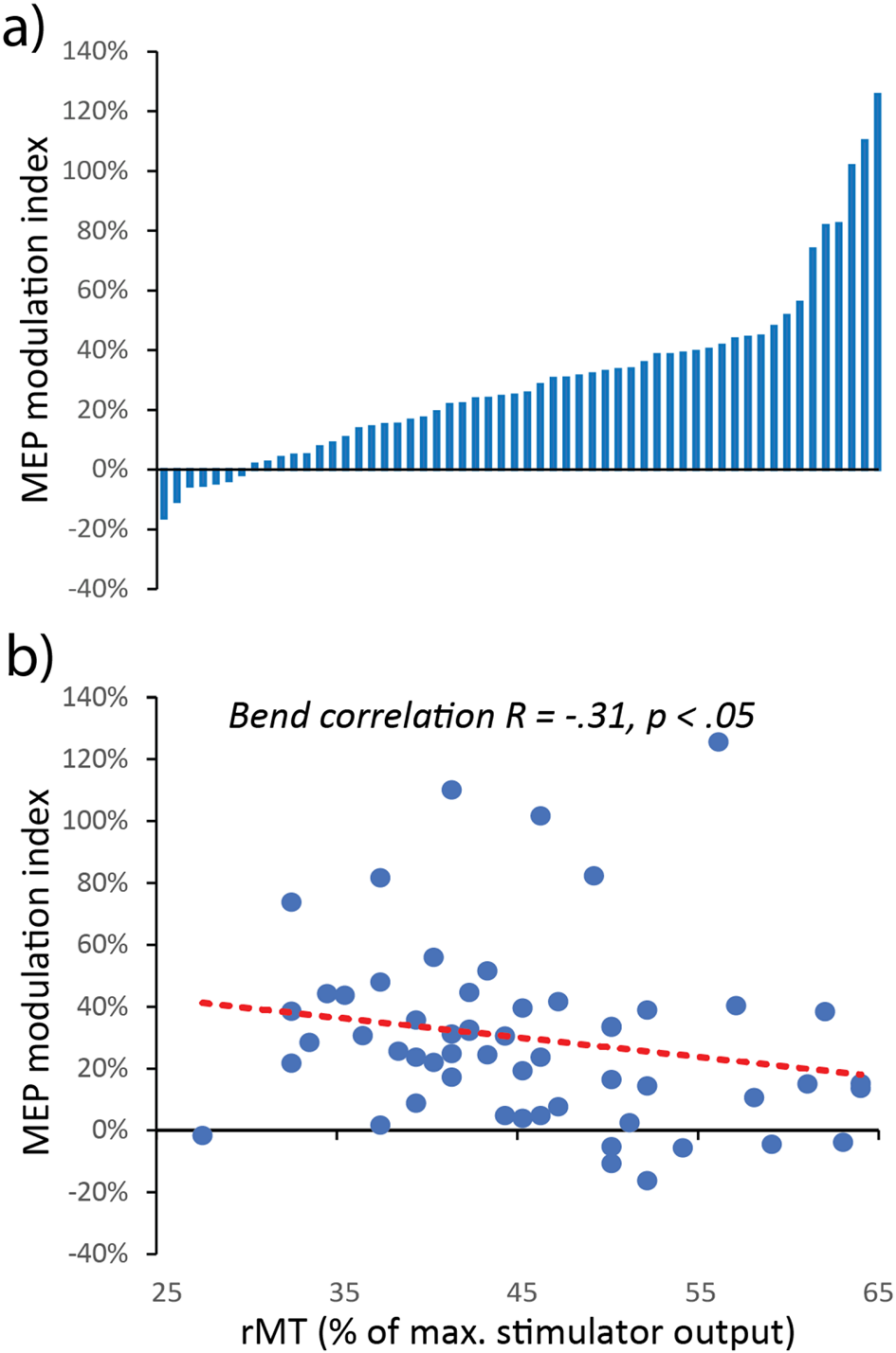
(**a**) Individual variability in the response to forward-ccPAS as shown by the distribution of individual MEP modulation indices computed as the percentage increase in the last epoch compared to the first epoch. (**b**) Relation between changes in MEPs and resting motor threshold (rMT) during forward-ccPAS.

Notably, the excitatory effect of forward-ccPAS was quite consistent across participants, although variable in magnitude. To assess inter-individual variability, we computed a MEP modulation index as the percentage increase of MEP amplitude in the last epoch compared to the first epoch [(last epoch − first epoch)/first epoch * 100]. Figure 2a shows that the vast majority of participants (87.5%) presented larger MEPs at the end of the protocol, 75% showed an increase of at least + 10% and 46% showed a consistent increase of at least + 30% in the last epoch. In contrast, only 3.6% of participants showed a reduction of approximately 10% in the last epoch.

Building on previous studies investigating predictors of TMS aftereffects^11,18^, we also tested whether individual’s rMT-a reliable global measure of motor excitability^31^-predicted differences in the magnitude of MEP increase during forward-ccPAS. We found that rMT significantly predicted the MEP modulation index (*Bend Correlation R* = − 0.31; *P* = 0.01; Fig. 2b), with participants with lower rMT showing the greater increase in corticomotor excitability.

In a control analysis, we checked whether there was an influence of the type of TMS machine, gender and motor activity carried out just before ccPAS and found no evidence supporting a role of these factors in modulating the strength of the ccPAS effect (Fig. S1).

Lastly, to provide more insights into the temporal features of the modulatory effects, we plotted the distribution of the single-trial MEPs (Fig. 1c). Importantly, during the administration of the 90 pulses in the forward ccPAS condition, we observed an increase in MEP size, accurately fitting a linear distribution (f(x) = 0.006*x + 1.002; R^2^ = 0.89).

**Figure 3:**
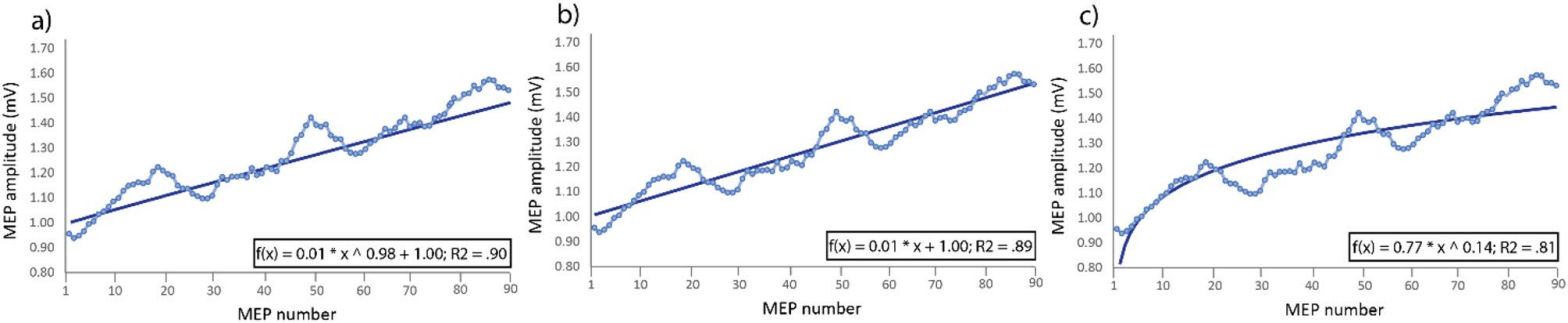
Fitting equations for single-trial MEPs distribution during forward-ccPAS: (**a**) two-term Power distribution; (**b**) linear distribution; (**c**) single-term power distribution.

We compared different fittings to establish which one better described changes in MEPs during forward-ccPAS (Fig. 3) and found several adequate equations fittings. The best fitting equation corresponded to a two-term power distribution (f(x) = 0.007 * x^0.977^ + 0.998; R^2^ = 0.90; Fig. 3a). However, such an equation is virtually corresponding to a simple linear distribution, which indeed proved to have an almost identical graph and R^2^ (Figs. 1c, 3b). Lastly, a single term power could also be used to adequately describe our results, although achieving a lower R^2^ (f(x) = 0.766 * x^0.141^; R^2^ = 0.81; Fig. 3c). The linear fitting, therefore, appeared to be the best accurate model and notably, the observed increase suggests that no clear plateau was reached by the end of stimulation (15 min). Moreover, although we observed no significant change across epochs during reverse-ccPAS, the last portion of the graph indicates a clear trend towards reduced corticomotor excitability.

## Discussion

We highlight the dynamics of changes in corticomotor excitability during ccPAS over PMv and M1. Our study shows a gradual increase in MEP amplitudes during forward-ccPAS, targeting the connection from PMv to M1, with continuous amplitude increase along the stimulation train. In contrast, reverse-ccPAS showed a trend toward inhibition at the end of the train. Thus, pattern of corticomotor excitability was not merely due to repeated stimulation of PMv or M1, and critically depended on the order between each pair of pulses over these two areas.

The gradual changes in MEP amplitudes that we observed are in line with prior work highlighting dose-dependent effects of TMS, and showing larger effects associated with increasing the number of pulses within a single session^32,33^ or along the number of sessions^34–36^. Interestingly, MEP increase during forward-ccPAS fitted a linear model and did not reach a plateau at the end of the train, raising the interesting issue of protocol duration: in most of the prior studies, the number of pulses in the ccPAS protocol have been arbitrarily set between 90 and 100^1–3,8–14^. Our findings of linear, dose-dependent MEPs increase during forward-ccPAS, suggest that increasing the number of paired-stimulations may induce more prominent plastic effects. This possibility is potentially relevant for clinical applications of ccPAS, when stronger alterations could be desirable. Yet, this should be directly tested as the relationship between additional doses and changes in excitability could evolve non-linearly^37,38^. Moreover, while our study suggests that the number of paired pulses could be a relevant variable to consider to increase ccPAS effectiveness, future studies could also test the role of frequency and intensity of stimulation, as these parameters are known to influence the effects of repetitive TMS^31,39^; moreover, implementing closed-loop state-dependent paradigms may offer additional specificity and efficiency benefits.

A growing literature shows that the effect of brain stimulation is highly variable across individuals^40–43^. In keeping, while most participants receiving forward-ccPAS showed a consistent increase in corticomotor excitability, the magnitude of the increase was variable across them. Notably, we found that the magnitude of MEP enhancement during forward-ccPAS was predicted by interindividual differences in rMT, with larger MEP enhancement associated with lower rMT. Because the rMT provides a measure of motor excitability^33^, these findings lend direct support to the notion that greater motor excitability is associated with higher sensitivity to associative plasticity^11,18^.

In our control analyses we further explored individual predictors of sensitivity to ccPAS manipulation by testing the influence of gender. These analyses suggest no influence of this factor (Fig. S1)﻿, in keeping with a prior TMS study testing STDP effects^44^. However, we did not assess the phase of the menstrual cycle of female subjects, and prior reports suggest a possible influence of ovarian hormones on motor system sensitivity to repetitive TMS^45^. Moreover, we tested young participants only, thus limiting the possibility to investigate the effect of age on STDP. Future studies should further explore inter-individual differences in responsiveness to ccPAS, and factors that account for such variability, such as age, gender or genetic polymorphisms, and, crucially, individual patterns of structural brain connectivity^44^; finally, because the present work has used a between subject design, it has not been possible to characterize whether each individual’s malleability to forward-ccPAS protocols and reverse-ccPAS protocols correlate. Despite these limitations, our study identifies a common and well-established neurophysiological parameter, namely the individual’s rMT, as a predictor of ccPAS sensitivity, expanding prior studies focusing on forward-ccPAS aftereffects^11^ and thus providing insights into the issue of individualized approaches to brain stimulation.

While we observed protocol-specific effects, with a clear increase in corticomotor excitability during forward-ccPAS and a tendency toward decrease in the last phases of reverse-ccPAS, our measure (MEPs) does not clarify the precise level at which plastic effects occur (e.g., cortico-cortical connections, M1 corticospinal neurons, or both). We did not include a control condition (i.e., a single-pulse stimulation of M1 to record unconditioned MEPs) interleaved with the protocol’s paired-stimulation, as such control stimulation could potentially interfere with ccPAS efficacy, by reducing the coherence of the repeated paired-stimulation-which is essential for STDP to occur^1–7^. Moreover, the bulk of available works, including ours, have limited their investigation to the left (dominant) hemisphere of right-handed participants and the malleability of the right hemisphere PMv-to-M1 pathway to ccPAS manipulation remains to be established^2,11,12,14^. However, prior ccPAS studies targeting interhemispheric and right-hemisphere motor and/or visual circuits have commonly reported results coherent with the notion of STDP^1,15,16,46^, similarly to studies testing the left hemisphere^2,11,12,14^; moreover, studies directly testing STDP-effects over the left and right M1 have commonly reported comparable long-term potentiation effects in the two hemispheres^47^.

Additionally, our study has only assessed one ISI between the two interested nodes, namely 8 ms; this specific timing was chosen based on previous results indicating it as the most effective to probe direct cortico-cortical connections between PMv and M1^29,30,48^. Indeed, prior studies have established that the most effective ISI for driving STDP with ccPAS corresponds to the most effective ISI to probe cortico-cortical connections^1–4,7–17^. Although we did not investigate further ISIs, our design allows to rule out that the increase of corticomotor excitability that we observed following forward-ccPAS was due to the mere stimulation of PMv and M1, as we observed no increase in excitability in the (control) reverse-ccPAS condition. However, in a previous ccPAS study targeting the PMv-M1 circuit^12^, we found that a ccPAS using a longer ISI between the pulses, based on long-latency cortico-cortical connectivity^49,50^, is also able to induce STDP-like effects. Thus, future studies should explore the comparative efficacy of ccPAS protocols informed by different timings and assess whether personalizing the ISI to match individual connectivity patterns could maximize ccPAS efficiency^51–53^.

Despite these limitations, we can conclude that ccPAS over the PMv-M1 circuit induces a consistent modulation of corticomotor excitability that gradually and linearly builds up already during protocol administration, and depends on stimulation parameters (i.e., order of the paired-pulse) and interindividual differences in motor excitability. All in all, our study suggests that MEP monitoring during STDP manipulation could provide insights into the malleability of the motor system and protocol’s effectiveness, and paves the way to the possibility to adopt real-time physiological monitoring during ccPAS for optimizing individual stimulation parameters in experimental and clinical settings.

## Material and methods

### Participants

A total sample of 109 right-handed healthy volunteers took part in this study after providing written informed consent. All were right-handed, based on the Edinburgh Handedness Inventory, had normal or corrected-to-normal vision and were naïve to the purpose of the study. All participants gave written informed consent prior to the study and were screened to avoid adverse reactions to TMS^31^ and exclude individuals with neurological disorders or subject to pharmacological treatment acting on the central nervous system. The study was carried out at the Centro studi e ricerche in Neuroscienze Cognitive, University of Bologna. The study was conducted in accordance with the ethical standards of the 1964 Declaration of Helsinki and approved by the Bioethics Committee of the University of Bologna (2.6/07.12.16). None of the participants reported adverse reactions or discomfort related to TMS.

The sample was randomly divided into two groups (Fig. 1a). The first group (N = 56, 36 females, mean age ± SD: 22.6 y ± 2.6) underwent premotor-motor ‘forward-ccPAS’: on each TMS pair, a conditioning pulse over PMv was administered immediately before M1 stimulation (ISI = 8 ms), so that the first TMS pulse (PMv) would elicit a cortico-cortical volley reaching M1 slightly before the second TMS pulse (M1), resulting in convergent M1 activation-optimal for inducing STDP^1–14^. A second group (N = 53, 30 females, mean age ± SD: 22.8 y ± 2.7) underwent ‘reverse-ccPAS’, having each M1 stimulation followed by PMv stimulation. This control condition allows us to rule out that any observed effects of forward-ccPAS may be ascribed to the mere repeated stimulation of PMv or M1, and critically depended on the order between each pair of pulses over these two areas.

Participants in this study were tested in further sessions before and after ccPAS. Specifically, 21 participants were tested in visuomotor dexterity and choice reaction tasks (results from this study have been already published^11^); 40 and 48 participants were tested in two further experiments testing the effect of ccPAS on imitation and M1 intracortical excitability, respectively. All three studies reported significant and coherent after-effects; these data will be presented in separate publications addressing different and independent research questions and focusing on ccPAS aftereffect. Because the ccPAS procedure was the same across the different experiments (see below), here, we pooled data from the three experiments together to increase sample size and drawn more robust conclusions regarding MEP changes during ccPAS administration.

### General experimental design

We administered ccPAS over PMv and M1; the protocol consisted of 90 pairs of TMS pulses over the two areas delivered at 0.1 Hz frequency^2,11,12,14^. Importantly, M1 stimulation was performed using suprathreshold TMS intensity. Thus, on each paired-stimulation we induced a motor-evoked potential (MEP) in the relaxed right first dorsal interosseous (FDI), allowing to track the emergence of changes in corticomotor excitability during protocol administration.

### ccPAS

TMS was administered using two 50-mm butterfly-shaped iron-branding coils. In both forward and reverse-ccPAS protocols, we administered 90 pairs of TMS pulses at a rate of 0.1 Hz for 15 min^1–3^. Each participant’s rMT was assessed using the established procedure as the minimum stimulator output intensity able to induce MEPs > 50 μV in 5 out of 10 consecutive trials^31^. In all participants, rMT was assessed immediately before the ccPAS protocol. In the forward-ccPAS protocol a first pulse was administered over the left PMv and the second pulse was administered over the left M1 with an ISI of 8 ms, so to activate short-latency PMv-to-M1 connections^20,29,30^. In the reverse-ccPAS protocol, instead, the order of stimulation was reversed, with the M1 pulse always preceding the one over PMv. In both groups, the PMv pulse intensity was set to 90% of the individual’s rMT while the M1 stimulation was adjusted to evoke ~ 1 mV MEPs^1–3^. TMS was performed using either two independent Magstim 200 (monophasic) stimulators (in 88 participants) or a Magstim 200 stimulator for PMv stimulation and a Magstim Rapid2 (biphasic) stimulator for M1 stimulation (see Supplementary Results).

### Brain localization

Coil positions were identified using established methods^11,12,49,50^ as detailed below. The left M1 was identified functionally as the FDI motor hotspot. To target M1, the coil was held at 45° to the sagittal midline inducing a posterior-to-anterior current direction in the brain^54^. The left PMv was identified using the SoftTaxic neuronavigation system (EMS, Italy). Skull landmarks (nasion, inion, and two pre-auricular points) and about 90 points providing a uniform representation of the scalp were digitized by means of the Polaris Vicra digitizer (Northern Digital INC, Ontario, CA). An individual estimated magnetic resonance image (MRI) was obtained for each subject through a 3D warping procedure fitting a high-resolution MRI template with the participant’s scalp model and craniometric points. The targeted an anterior sector of the PMv at the border with the posterior part of the inferior frontal gyrus using the following Talairach coordinates: x =  − 52, y = 10, z = 24. These coordinates were obtained by averaging the coordinates reported in previous studies^55–59^; these studies showed that stimulating this ventral frontal site affected planning, execution and perception of hand actions, confirming the functional relevance of the PMv site. The selected PMv coordinates are consistent with those used in previous ccPAS^2,13,14^ and dual-site TMS studies targeting the PMv-to-M1 circuit^16–48^. The coil over PMv was placed at ~ 45° to the midline to induce a ventro-lateral to medio-posterior current^49,50,55^.

The scalp locations that corresponded best to left M1 and left PMv coordinates were identified and marked with a pen. Then, the SofTaxic Navigator system was used to estimate the projection of all targeted scalp positions on the brain surface, confirming correct coil placement for all the sites. Across the forward-ccPAS and reverse-ccPAS groups, the estimated Talairach coordinates for the left M1 were (mean ± S.D.): x = –30.6 ± 5.5, y = –17.1 ± 6.8, z = 59.6 ± 3.9; for the left PMv were: x = –53.4 ± 1.8, y = 10.1 ± 1.7, z = 23.4 ± 1.9.

### Data analysis

Peak-to-peak MEP amplitudes induced by M1 stimulation in the FDI muscle were automatically extracted from EMG signals using a custom MatLab code (MathWorks, USA) and measured in mV. Trials showing EMG activity 100 ms prior to TMS were discarded from further analysis (4.7%). The 90 MEPs recorded during the ccPAS were divided into 6 epochs of 15 MEPs each, and the mean MEP amplitude in each epoch was transformed using the formula Log10(value + 1) to address lack of normality. These data were analyzed using a Protocol x Epoch ANOVA, whose results are reported in the main text.

To explore predictors of MEP changes, we first calculated a modulation index for each subject as the MEP amplitude in the last epoch divided by the MEP amplitude in the first epoch. Then, we computed robust correlations between such MEP modulation index and individual’s rMT using MatLab Toolbox^60^.

## Supplementary Information

Supplementary Information.

## Data Availability

The data that support the findings of this study are available from the corresponding author (AA), upon request.
