# Supplementary material for: Gradual enhancement of corticomotor excitability during cortico-cortical paired associative stimulation
Source: Sci Rep. 2022 Aug 29;12:14670. doi: 10.1038/s41598-022-18774-9 (PMC9424198; doi:10.1038/s41598-022-18774-9)
Supplement: Supplementary file 1 — Supplementary Information. [file 41598_2022_18774_MOESM1_ESM.docx]

**Gradual enhancement of corticomotor excitability during cortico-cortical paired associative stimulation**

Sonia Turrini, Francesca Fiori, Emilio Chiappini, Emiliano Santarnecchi, Vincenzo Romei, & Alessio Avenanti

**Supplementary information**

**Supplementary Results**

|  | **Epoch 1** | **Epoch 2** | **Epoch 3** | **Epoch 4** | **Epoch 5** | **Epoch 6** |
| --- | --- | --- | --- | --- | --- | --- |
| **Forward-ccPAS** | 1.05 ± 0.36 | 1.15 ± 0.51 | 1.20 ± 0.53 | 1.34 ± 0.56 | 1.37 ± 0.58 | 1.51 ± 0.62 |
| **Reverse-ccPAS** | 1.14 ± 0.55 | 1.14 ± 0.69 | 1.19 ± 0.61 | 1.16 ± 0.76 | 1.23 ± 0.81 | 1.12 ± 0.82 |

**Table S1.** Raw MEP amplitudes in mV ± standard deviation.

***Influence of study design or gender on ccPAS efficacy***

Because the present work pools together data from three studies that have used the ccPAS protocol over the same PMv-M1 circuit but had different general experimental designs, it would be theoretically possible that different pre-ccPAS test blocks might have influenced the activation status of the selected PMv-to-M1 pathway, resulting in it being more or less malleable and responsive to ccPAS between the three studies. For example, in Study 1 pre-ccPAS test blocks involved neurophysiological assessment while participants remained at rest, whereas in Study 2 and 3 participants actively performed motor tasks (imitation and manual dexterity tasks, respectively). Moreover, while participants from Study 1 and 2 were tested using two monophasic Magstim 200 stimulators, participants in Study 3 were tested using a monophasic Magstim 200 stimulator for PMv stimulation and a biphasic Magstim Rapid2 stimulator for M1 stimulation (as reported in the study of Fiori et al. [1]). Because the biphasic stimulator induces a main current spread in opposite direction relative to the monophasic stimulation, the coil over M1 was rotated to induce a posterior-to-anterior current spread in all participants.

To ensure that such differences were not to contaminate the reported increase in corticomotor excitability during administration of forward-ccPAS, we computed the slope of the MEP increase across the 90 paired-pulses, separately for each of the three studies, and compared them through a 1-way ANOVA. The analysis was not significant (F_2,53_ = .37, p = .68, Figure S1, panel a), indicating similar slopes across Study 1-3. Thus, MEP enhancement observed during forward-ccPAS was comparable in the two subgroups of participants performing motor tasks before ccPAS (Study 2 and 3) relative to the subgroup of participant tested at rest (Study 1). Moreover, results suggest comparable findings when using two monophasic stimulators (Study 1 and 2) relative to a monophasic and biphasic stimulator (Study 3). The same results were obtained when using the MEP modulation index (F_2,53_ = .24, p = .78).

We subsequently adopted the same method to test for any gender related differences in individual responsiveness to ccPAS, comparing our female and male participants; both the slope (F_1,54_ = .001, p = .96, Figure S1, panel b) and the MEP modulation index (F_1,54_ = 1.22, p = .27) were comparable between them.

Finally, to ensure the absence of any interaction between gender related differences and the three different study designs we ran two ANOVAs with factors gender (male, female) and experimental design (Study 1, Study 2 and Study 3) on both the slope of the MEP increase and MEP modulation index. For both ANOVAs, no main effects or interactions reached significance (all Fs < 1.08, all p > .31)


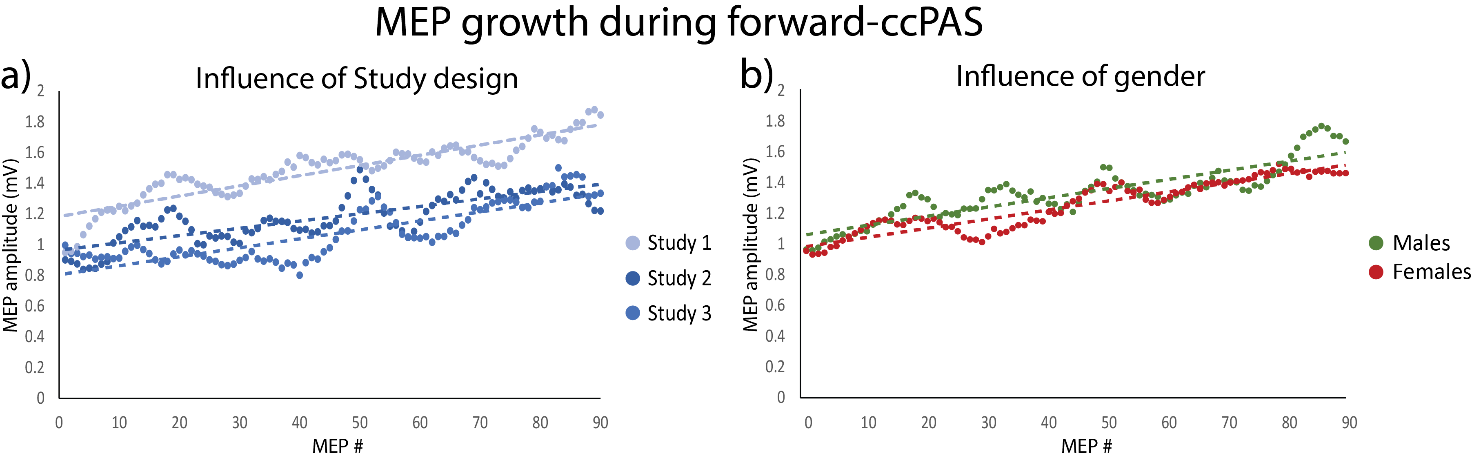


**Figure S1.** Influence of Study design (a) and gender (b) on MEP growth during forward-ccPAS.

**References**

1. Fiori, F., Chiappini, E., & Avenanti, A. (2018). Enhanced action performance following TMS manipulation of associative plasticity in ventral premotor-motor pathway. *Neuroimage* **183**, 847–858.
